# Petasites for Migraine Prevention: New Data on Mode of Action, Pharmacology and Safety. A Narrative Review

**DOI:** 10.3389/fneur.2022.864689

**Published:** 2022-04-26

**Authors:** Jürgen Borlak, Hans-Christoph Diener, Johanna Kleeberg-Hartmann, Karl Messlinger, Stephen Silberstein

**Affiliations:** ^1^Centre for Pharmacology and Toxicology, Hannover Medical School, Hannover, Germany; ^2^Institute for Medical Informatics, Biometry and Epidemiology (IMIBE), University Duisburg-Essen, Essen, Germany; ^3^Institute of Physiology and Pathophysiology, University of Erlangen-Nürnberg, Erlangen, Germany; ^4^Jefferson Headache Center, Thomas Jefferson University, Philadelphia, PA, United States

**Keywords:** migraine, petasins, migraine prevention, mode of action, pharmacology, safety

## Abstract

Petasins are the pharmacologically active ingredients of butterbur and of therapeutic benefit in the treatment of migraine and tension headaches. Here, we summarize the pharmacology, safety and clinical efficacy of butterbur in the prevention of migraine attacks and present new data on its mode of action. We review published literature and study reports on the safety and clinical efficacy of the butterbur root extract Petadolex® and report new findings on petasins in dampening nociception by desensitizing calcium-conducting TRP ion channels of primary sensory neurons. Importantly, butterbur diminishes the production of inflammatory mediators by inhibiting activities of cyclooxygenases, lipoxygenases and phospholipase A2 and desensitizes nociception by acting on TRPA1 and TRPPV1 ion channels. It inhibits the release of calcitonin-gene related peptide (CGRP) of meningeal afferents during migraine attacks. We also evaluated the safety of a butterbur root extract in repeated dose studies for up to 6 months. A no-observable-adverse-effect-level at 15-fold of the maximal clinical dose (3 mg/kg/day MCD) was established for rats. At supratherapeutic doses, i.e., 45–90-fold MCD, we observed bile duct hyperplasia, and mechanistic studies revealed regulations of solute carriers to likely account for bile duct proliferations. Additionally, liver function tests were performed in cultures of primary human hepatocytes and did not evidence hepatotoxicity at therapeutic butterbur level and with migraine co-medications. Lastly, in randomized, double-blinded and placebo-controlled trials with Petadolex® migraine attack frequency was reduced significantly at 150 mg/day, and no relevant abnormal liver function was reported. Together, butterbur is effective in the prevention of migraine attacks by blocking CGRP signaling.

## Introduction

For its medicinal properties the perennial herb butterbur (Petasites hybridus) has been used for centuries (1–5). Butterbur is a member of the Asteraceae family of plants and grows in many parts of the world. So far 18 species of this genus have been identified (6), and traditional indications include the treatment of migraine and pain associated with tension headaches, stomach pain, allergic rhinitis, asthma and spasms of the gastro-intestinal tract (1, 7–10). Petasins are the pharmacologically active ingredients of butterbur and are defined as sesquiterpenes, i.e., esters of petasol and angelic acid. Notwithstanding the various species differ in the amount and composition of sesquiterpenes with implications for its therapeutic use.

Petasins are responsible, at least in part, for the anti-inflammatory effects of butterbur and are related to the ability to inhibit prostaglandin and leukotriene biosynthesis (11, 12). Given the importance of inflammation to the pain of migraine, the anti-inflammatory properties of petasins provide a rationale for the use of butterbur in the treatment of migraine attacks (13).

Among the various butterbur extracts Petadolex® is a well-established herbal remedy for the prevention of migraine attacks. It is produced from the rhizome of Petasites hybridus and is the only butterbur extract that was extensively evaluated for safety and clinical efficacy in the preventive treatment of migraine. Petadolex® was introduced 1972 in Germany and 1998 in the USA, and pharmacological research identified petasins as active ingredients.

Here we review the current knowledge on butterbur and specifically petasins for their use in the preventive treatment of migraine. We begin with a discussion of the clinical efficacy of the Petasites hybridus root extract Petadolex® followed by a general discussion of butterbur composition. Next we consider a number of potential mechanisms that could account for therapeutic benefits in migraine; this includes anti-inflammatory effects in addition to inhibition of calcitonin gene-related peptide (CGRP) release (14). CGRP is a peptide of paramount importance in nociceptive pain transmission. We also review the limited information on the pharmacokinetics, *in vitro* drug interactions, toxicology, genotoxicity, regulation of bile salt transporters and finally tie up all the information for a better understanding of butterbur's therapeutic benefit in the prophylaxis of migraine.

## Methods

We performed a PubMed literature search using the key word butterbur in March 2022. This revealed 311 entries over a period of 70 years. Interestingly, we noted a significant surge in research activities with 22 entries alone in 2021. Indeed about 85% of PubMed entries started from the year 2000. Subsequently, we refined the search strategy using the following key words:

1. Petasites hybridus root extract chemical analysis.
2. Butterbur neuroprotection.
3. Butterbur root extract clinical trials.
4. Butterbur clinical trials migraine.
5. Butterbur root extract pharmacology and toxicity.
6. Butterbur mechanism of action.
7. Petasites mode of action.
8. Butterbur extract pharmacology and safety.

We considered study reports from University laboratories and especially doctoral theses in addition to study reports of contract research organizations who were commissioned by the Petadolex® vendor Weber und Weber GmbH, Germany to perform research on butterbur root extracts. The study reports were submitted to the authors.

In regards to safety data and next to published clinical trials we considered preclinical data especially of Good Laboratory Practice (GLP) compliant investigations performed by contract research organization (reports were submitted to the authors).

The acceptance criteria for data inclusion in this narrative review are (a) published reports in peer-reviewed journals in English or German and cited in PubMed, (b) information retrievable as doctoral thesis from public repositories and University archives, (c) personal communications, if the information is pertinent to an understanding of butterbur modes of action and its therapeutic benefit in the treatment of migraine.

In addition, we evaluated the evidence level of butterbur's mode of action and clinical efficacy by considering the following criteria: Evidence level 1 = individual published reports in PubMed cited peer reviewed journals; evidence level 2 = confirmative reports from independent investigators across multiple centers in PubMed cited peer reviewed journals; evidence level 3 = at least three independent PubMed cited reports in peer reviewed journals confirming the initial claim.

Given that Petadolex® is the only butterbur extract investigated for migraine in clinical trials we summarize the evidence for its therapeutic efficacy but due to lack of information are unable to compare it to other extracts such as Petasites hybridus leaf extracts or any other Petasites herbal preparations. For its proven anti-inflammatory mode of action we also refer to studies on allergic rhinitis and asthma in clinical trials.

**Figure 1:**
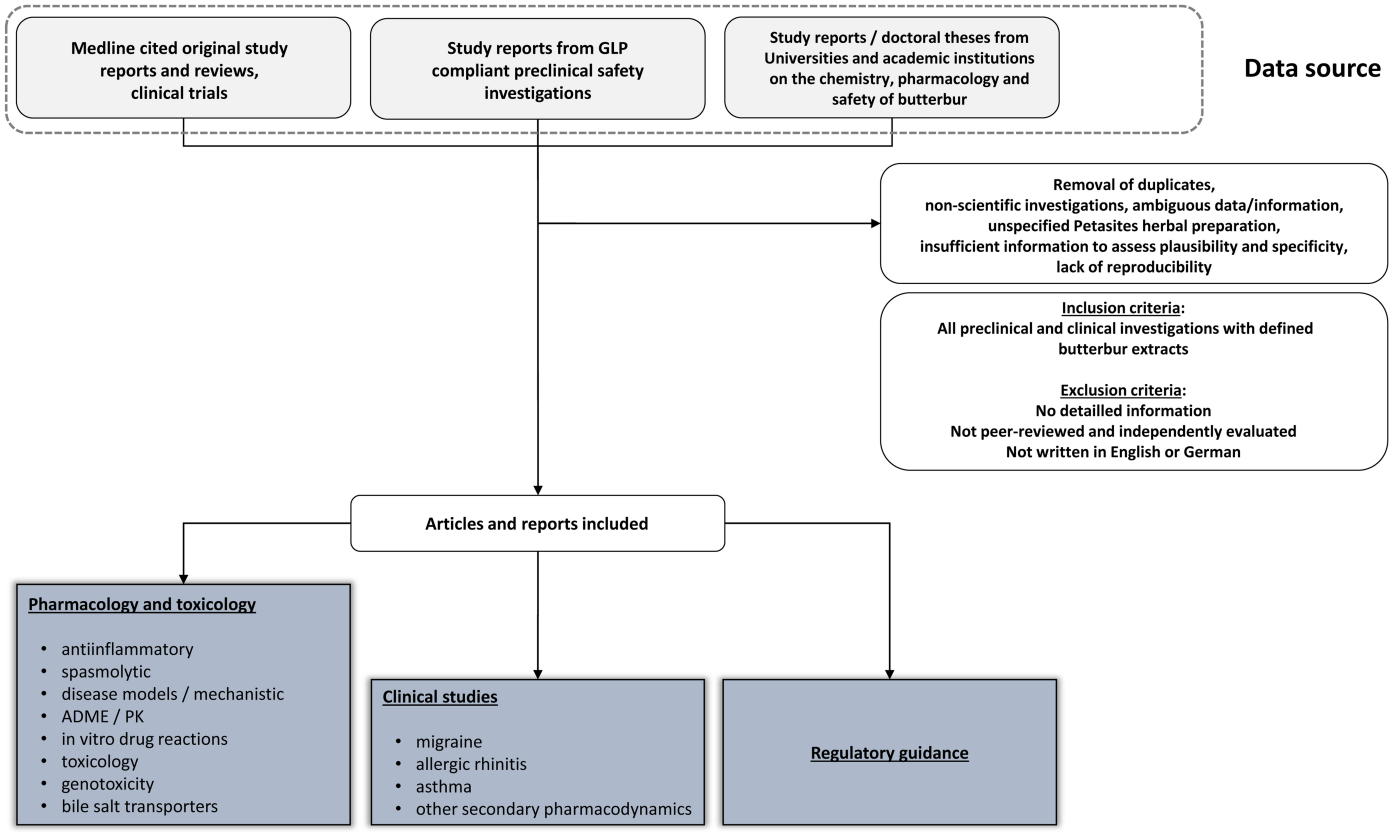
Quorum flow chart describing the data sources and inclusion criteria for assessment.

Shown in Figure 1 is the search strategy in retrieving relevant information of this narrative review.

## Butterbur's Composition and Therapeutic Benefits

### Composition of Petasites Hybridus Extracts

Extracts are prepared from the rhizome and/or leaves of butterbur. Its chemical analysis revealed petasins (=sesquiterpenes, i.e., esters of petasol and angelic acid) as major constituents in addition to essential oils and pyrrolizidine alkaloids (PAs). The PAs are minor components; however, the PA content of leaves and roots (=rhizome) differs (15) with >90% of alkaloids found in the root extracts. Other constituents are the isomeric oxopetasan esters (16), sesquiterpene hydrocarbons, bakkenolides, petasitene and pethybrene; the latter is a constituent of the essential oil fraction (17).

Pharmacological research identified petasins as active ingredients but extracts differ in the amounts of petasins and their structural analogs, i.e., iso- and neopetasin as well as S-petasin, iso-S- and neo-S-petasin (18). To date >20 different eremophilanes (=bicyclic sesquiterpenoids) have been reported (19–21). Research identified furanoeremophilanes in extracts of Petasites hybridus giving rise to distinct chemotypes, i.e., the petasin or furan chemovars of butterbur.

### Anti-inflammatory Effects

The anti-inflammatory activity of Petasites hybridus extracts is attributed to its sesquiterpene ester components, such as petasin and isopetasin. Butterbur decreases the production of the inflammatory mediators prostaglandin E2 (PGE2), leukotriene B4 (LTB4) and cysteinyl-leukotrienes (LTs), in animal and human cellular system, as well as purified enzyme preparations (11). In addition, asthmatic patients were shown to benefit from butterbur's anti-inflammatory activity whilst on steroids (22).

Petasin blocks intracellular calcium influx into neutrophils and eosinophils and inhibits leukotriene biosynthesis (11). Additionally, the Ca-antagonistic properties of petasins were proven in aortic ring preparations from rats. Here, petasins inhibited L-type voltage-dependent Ca^2+^ channel (VDCC) activity in a concentration dependent manner, and the petasins-induced vasorelaxation was confirmed in blood vessels of spontaneous hypertensive rats (23). However, a significant interaction between petasins and the dihydropyridine binding sites of L-type VDCC has not been established as yet (23). In an earlier study, the same investigator demonstrated Ca^2+^ channel blocking effect of S-isopetasin in rat aortic smooth muscle cells (24). Additionally, S-isopetasin isolated from Petasites formosanus depresses cardiac contraction and intracellular Ca^2+^ transients in adult rat ventricular myocytes (25). Taken together, the findings are particularly relevant as a recent study highlights an association of migraine with the incidence of hypertension after menopause (26). Obviously the relationship between migraine and high blood pressure is complex; nonetheless the calcium channel blocking activity of petasins might be of therapeutic benefit even though the clinical evidence regarding the use of Ca-channel blockers in the prophylactic treatment of migraine is conflicting.

Voltage clamp studies with a mouse neuroblastoma and rat glioma hybrid cell line also revealed S-petasin of Petasites formosanus to interact directly with L-type Ca^2+^ channels in NG108-15 cells (27) while studies with Xenopus laevis oocytes, which express presynaptic Ca(v)2.1 channels, confirmed the Ca(v)2.1-inhibitory properties of petasins (28). The findings are relevant for an understanding of butterbur's mode of action especially since voltage-gated Ca^2+^ channels are of critical importance in the transmission of nociceptive information (29).

Additionally, butterbur inhibits cyclooxygenase-2 (COX-2) activity (30). The inhibition is independent of the petasin and isopetasin content. This suggests that the therapeutic effect of butterbur on cyclooxygenase inhibition may not result from a single constituent of the extract alone (30).

**Figure 2:**
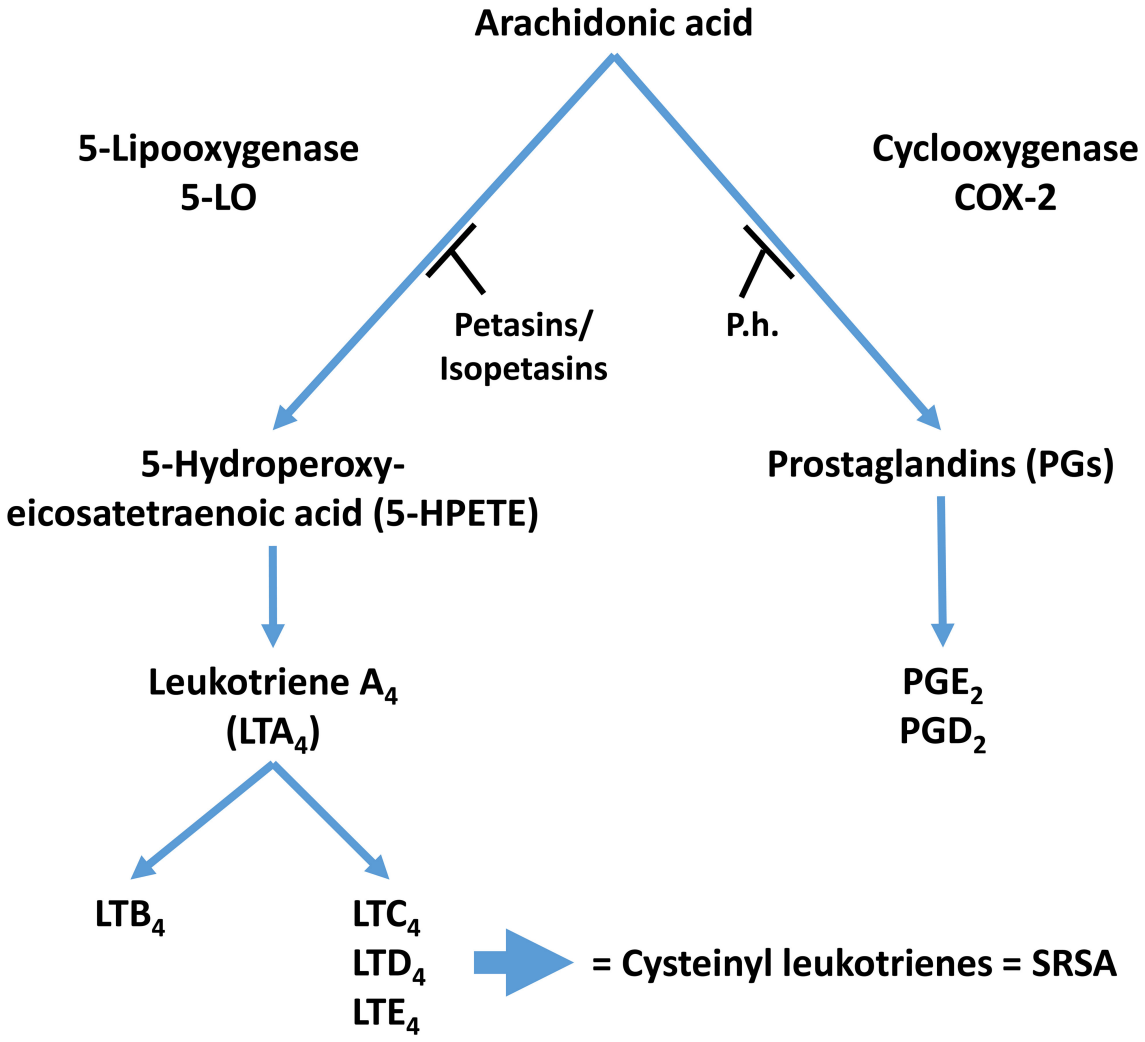
Proposed mechanism of action for Petasites hybridus extracts. Inhibition of 5-LO activity by Petasins and inhibition of COX-2 by butterbur extracts (T, Inhibition; LT, leukotriene; P.h., Petasites hybridus extract; SRSA, slow reacting substances of anaphylaxis).

Petasin, isopetasin and neopetasin inhibit leukotriene biosynthesis in human blood cells (31). Petasin blocks eosinophil cationic protein release from eosinophils and platelet-activating factor- and/ or complement factor 5a-induced increases in intracellular Ca^2+^. Petasin also inhibits phospholipase A2 activity and 5-lipoxygenase translocation from the cytosol to the nucleus in stimulated eosinophils, therefore suggesting that petasins may block different intracellular signaling molecules (11). This leads to the notion that petasins may block earlier signaling events initiated by G protein-coupled receptors in granulocytes, including phospholipase C(beta) activity (11). Consistent with the importance of the inflammatory response in migraine attacks, phospholipase C activity is increased in cerebrospinal fluid of migraine patients (32). A summary of the anti-inflammatory activity of petasins is shown in Figure 2.

The anti-inflammatory activities of petasins differ in various experimental systems. Bickel et al. (12) reported that isopetasin but not petasin inhibited leukotriene biosynthesis in stimulated peritoneal macrophages of inbred NMRI-mice. Other investigators found petasin, isopetasin and neopetasin to inhibit leukotriene synthesis in diverse cellular systems (11, 31). For instance, petasites extract Ze 339 inhibited allergen-induced Th2 responses (IL-4, IL-5, and RANTES) and therefore airway inflammation in mice (33). Alike, the suppressive effects of Petasites japonicus extract on ovalbumin-induced airway inflammation was demonstrated in a mouse model of asthma (34), and similar anti-allergic effects were noted for ovalbumin-sensitized rats following treatment with bakkenolides (=sesquiterpene butyrolactones) which were isolated from the rhizome of Petasites tricholobus (35). Indeed, bakkenolide B inhibited lipopolysaccharide-induced pro-inflammatory cytokines via activated protein kinase (AMPK) / nuclear factor erythroid 2-related factor 2 (Nrf2) signaling in microglia (36, 37), and the anti-allergic and anti-inflammatory effects of bakkenolide B were independently confirmed in an ovalbumin-induced asthma model in which bakkenolide B strongly inhibited the accumulation of eosinophils, macrophages and lymphocytes in bronchoalveolar lavage fluid (38). There are further independent reports confirming petasins to suppress ovalbumin-induced airway inflammation (34, 35). For instance, the type I anti-allergic property of Japanese butterbur extract was reported for its mast cell degranulation inhibitory ingredients (39). More recently two new components in the leaves of Petasites japonicas were identified, i.e., petasitesin A and cimicifugic acid D which inhibited PGE2 and nitric oxide (NO) production in the RAW 264.7 macrophage cell line (40).

Additional anti-inflammatory effects of petasins are linked to inhibition of phospholipase C activity while isopetasin and neopetasin block 5-lipoxygenase activity. Note, 5-lipoxygenase is of key importance in neuro-inflammation, and the production of lipid mediators in the inflammatory responses. The pharmacological studies of Fiebich et al. (30) demonstrated inhibitory effects of Petasites hybridus extract in isolated COX-2 enzyme preparations as well as in microglial cells with impaired expression of COX-2 enzymes. This suggests that extracts of Petasites hybridus may exert inhibitory effects on PGE2 production in microglial cells by at least two different mechanisms, i.e., inhibition of COX-2 activity (i) and inhibition of COX-2 expression (ii). The furan chemovar of Petasites hybridus displayed a comparable dose-response relationship which suggests that both chemovars are equally pharmacologically effective (30). Thomet et al. (11) investigated the Petasites hybridus leave extract Ze339 and reported that petasin inhibits calcium flux and leukotriene production in cultures of human monocytes. Both the extract and petasin alone suppressed platelet-activating factor and the anaphylatoxin C5a mediated Ca^2+^ increases in eosinophils and neutrophils but the pharmacological 5-lipoxygenase inhibitor Zileuton had no effect. This points to petasin-mediated effects on leukotriene production that may arise from G protein-coupled receptor mediated phospholipase C(beta) signaling events in granulocytes (11).

Anti-inflammatory effects of the butterbur extract Petadolex® were also demonstrated in various *in-vitro* systems, e.g., swine leucocytes (41, 42), with inhibition of 5-lipoxygenase and selective inhibition of COX-2 (41, 43). Petasites root extracts inhibited dose-dependently lipopolysaccharide (LPS)-induced PGE2 release and p42/44 MAPK activation in primary rat microglial cells. Repression of COX-2 expression was also confirmed at the mRNA and protein level (30). Petadolex® inhibited the laurine sulfate-induced vascularization in the chicken embryo HET-CAM assay, again supporting an anti-inflammatory activity (44).

A murine macrophage study showed that petasin may antagonize the inhibition of isopetasin and oxopetasin on leukotriene E4 (LTE4) production (12).

**Figure 3:**
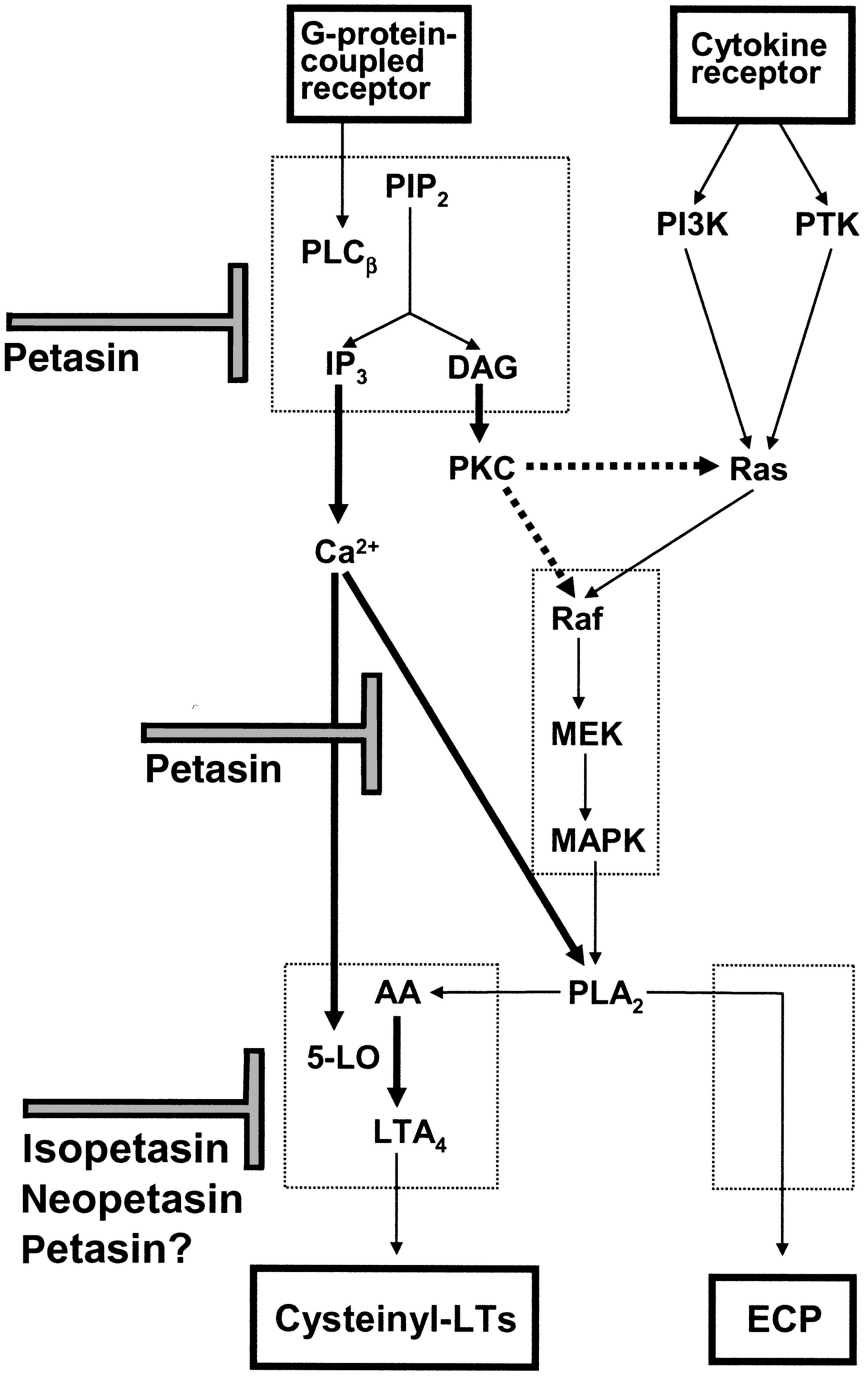
Scheme for the proposed mechanism of action of petasin, isopetasin and neopetasin in eosinophils. Agonist mediated intracellular calcium mobilization, ECP release and cysteinyl-LT production are inhibited by petasins and other constituents of Petasites hybridus extracts [taken from Thomet et al. (31)].

Taken together, butterbur extracts are anti-inflammatory, and the various studies provide a rationale for its traditional use. A summary of its different pharmacological activities is shown in Figure 3.

### Petasins and Neuroprotection

Several experimental studies confirmed the potential of petasins to be neuroprotective. Among the sequiterpenes bakkenolides were shown to protect neurons against cerebral ischemic injury by inhibiting activation of nuclear factor-κB (45). Note, NF-κB is a critical mediator of the inflammatory responses (46). A further example relates to bakkenolide-VI (1) isolated from Petasites tatewakianus which conferred neuroprotection in primary neuron cultures exposed to oxygen-glucose deprivation and oxidative insults (47). Bakkenolides from Petasites tricholobus are also neuroprotective, and the effects are related to its antioxidant activities (48). Alike, petaslignolide A, a furfuran lignan isolated from butanol fraction of Petasites japonicus is neuroprotective against oxidative damage as demonstrated in the brain of mice challenged with kainic acid (49). In the same animal model the neuroprotective effect of butterbur and rough aster (Aster scaber) was confirmed. The combination of both herbs delayed the onset time of behavioral signs and significantly preserved cytosolic glutathione peroxidase and glutathione reductase activities and therefore glutathione homeostasis (50). Additionally, the neuroprotective activities of sesquiterpenes isolated from Petasites japonicus were evaluated against cobalt chloride (CoCl2)-induced neuronal cell death in the human dopaminergic SH-SY5Y cell line. Among the newly identified compounds five displayed neuroprotective activity as judged by the MTT cell viability assay (51).

More recently, the AMPK/Nrf2 signaling pathway is the focus of research and petatewalide B, i.e., a bakkenolide-type sesquiterpine, alleviates oxygen-glucose deprivation/reoxygenation-induced neuronal injury via activation of the AMPK/Nrf2 signaling pathway in the human-derived neuroblastoma cell line SH-SY5Y (52).

### Antioxidant Activity

In a recent review the antioxidant compounds obtained from leaves and flower buds of Petasites japonicus were summarized (53), and the following constituents with proven antioxidant activity were identified: Quercetin 3-O-β-d-glucoside, quercetin 3-O-β-d-6”-O-acetylglucoside, rutin, caffeic acid, petasiformin A, petaslignolide A, chlorogenic acid, fukinolic acid, 3,5-dicaffeoyl quinic acid, 3,4,5-tricaffeoyl quinic acid and the flavonol kaempferol. Similarly, the chemical analysis of a methanol extract of leaves and roots of Petasites japonicus identified 5-caffeoylquinic acid, fukinolic acid, 3,5-di-O-caffeoylquinic acid, quercetin-3-O-(6”-acetyl)-β-glucopyranoside, 4,5-di-O-caffeoylquinic acid and kaempferol-3-O-(6”-acetyl)-β-glucopyranoside as antioxidants. Moreover, the leaves, stems and roots of Korean Petasites japonicus are a source of antioxidants especially 3,5-dihydroxy-7,3',4',5'-tetramethoxy flavanonol hydroxyl feruloyl glucoside, dicaffeoylquinic acid, naringenic hexoside, luteolin-7-O-[6'-dihydrogalloyl]-glucosyl-8-C-pentosyl-glucoside, liquiritin, 3,4-di-O-caffeoylquinic acid, 1,3-O-dicaffeoylquinic acid hexoside, kaempferol-3-O-acetylglucoside and chrysoeriol-methyl ether. Undoubtedly, butterbur is a rich source of antioxidants, and a recent study demonstrated butterbur extract powder of Petasites hybridus to ameliorate liver injury in ovalbumin-induced liver hypersensitivity reactions in Swiss albino male mice. Indeed, butterbur significantly decreased liver enzymes and oxidative stress and reduced the expression of markers for inflammation and fibrosis of liver tissues as shown by histopathology (54). Another recent report highlights the antioxidant activity of Petasites japonicus maxim. flower buds extracts that were given to ICR mice, i.e., a strain of albino mice, prior to iron injection (55). The extract significantly suppressed plasma thiobarbituric acid reactive substance (TBARS) production. TBARS and triglyceride concentrations in plasma of C57BL/6 were significantly decreased when the extract was given to mice on a high fat diet (55). Further examples of butterbur antioxidant activities include the identification of a furofuran lignin that was isolated from methanolic extracts of leaves of Petasites japonicas (56). The compound displayed antioxidant activity in DPPH radical scavenging assay and reduced seizure in kainic acid-treated mice. Conversely, an earlier report indicated adverse effects of Japanese butterbur leaves (Petasites japonicus, family Compositae) in the liver of male F344/DuCrj rats. Here, an acetone extract of the butterbur leaf powder caused increased TBARs activities in liver homogenates (57).

Given the relationship between inflammation and oxidative stress the identification of two new aryltetralin lactone lignans, i.e., petasitesins A and B are noteworthy, and the expressions of inducible nitric oxide synthase (iNOS) and COX-2 were inhibited in a macrophage cell line by both petasitesins (40).

### Petasins Desensitizes Nociception by Acting on TRP Channels of Primary Sensory Neurons

Although the clinical efficacy of Petadolex® in migraine therapy was reported nearly two decades ago (58), only recently it was shown that isopetasin desensitizes nociception by acting on cation transduction channels of the type transient receptor potential ankyrin 1 (TRPA1) (59). The effects of isopetasin were investigated by single-cell calcium imaging and patch-clamp recordings in human and rodent TRPA1-expressing cells as well as neurogenic motor responses in isolated urinary bladders (59). Additionally, the release of CGRP from mouse spinal cord was studied, and isopetasin markedly desensitized currents evoked by allyl isothyocyanate (AITC, TRPA1 channel agonist) or capsaicin, an agonist at transient receptor potential channels of vanilloid type type 1 (TRPV1), in rat trigeminal neurons.

Alike, the CGRP release from mouse central terminals of primary sensory neurons was significantly reduced. Therefore, petasins dampened nociception by de-sensitizing calcium-dependent TRP ion channel activity following nerve depolarization and impaired CGRP release from dense-core vesicles.

**Figure 4:**
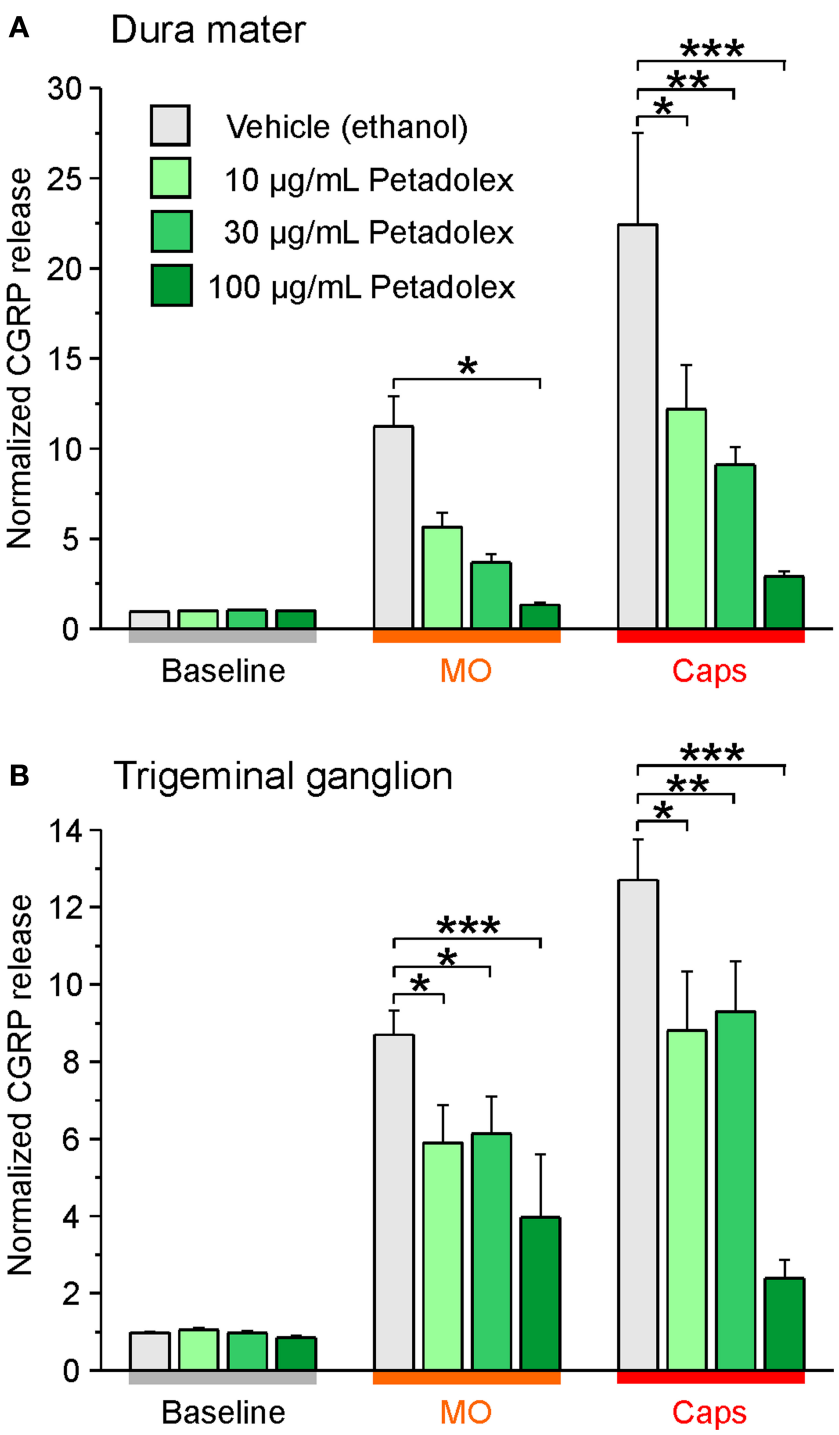
The butterbur root extract Petadolex® inhibits CGRP release from the dura mater **(A)** and trigeminal ganglia **(B)** of Wistar rats. Sagittally sectioned skulls freed of brain tissue but with adhering dura mater were incubated with butterbur root extract at concentrations of 10, 30 and 100 μg/mL for 1 h (*N* = 6 independent experiments). Data were compared to vehicle control treatments (ethanol, *N* = 12 independent experiments). Stimulation with 5 x 10^−4^ M mustard oil (MO) and 5 x 10^−7^ M capsaicin (Caps) evoked CGRP release reduced by Petadolex® pre-treatment; *p < 0.05, **p < 0.005, ***p < 0.001, ANOVA and LSD *post-hoc* test.

Independent studies confirmed impaired CGRP release from meningeal afferents of hemisected rodent head preparations by butterbur extracts (60). Specifically, Kleeberg-Hartmann et al. (14) stimulated CGRP release from the dura mater with the TRPA1 agonist allylisothiocyanate (mustard oil) and the TRPV1 agonist capsaicin (Figure 4A) for 5 min with physiological solution for baseline recordings. The CGRP release was reduced after incubation with the butterbur extract Petadolex® (Figure 4A). Likewise, pre-incubation of the trigeminal ganglion with Petadolex® reduced CGRP release in response to mustard oil and capsaicin stimulation (Figure 4B).

**Figure 5:**
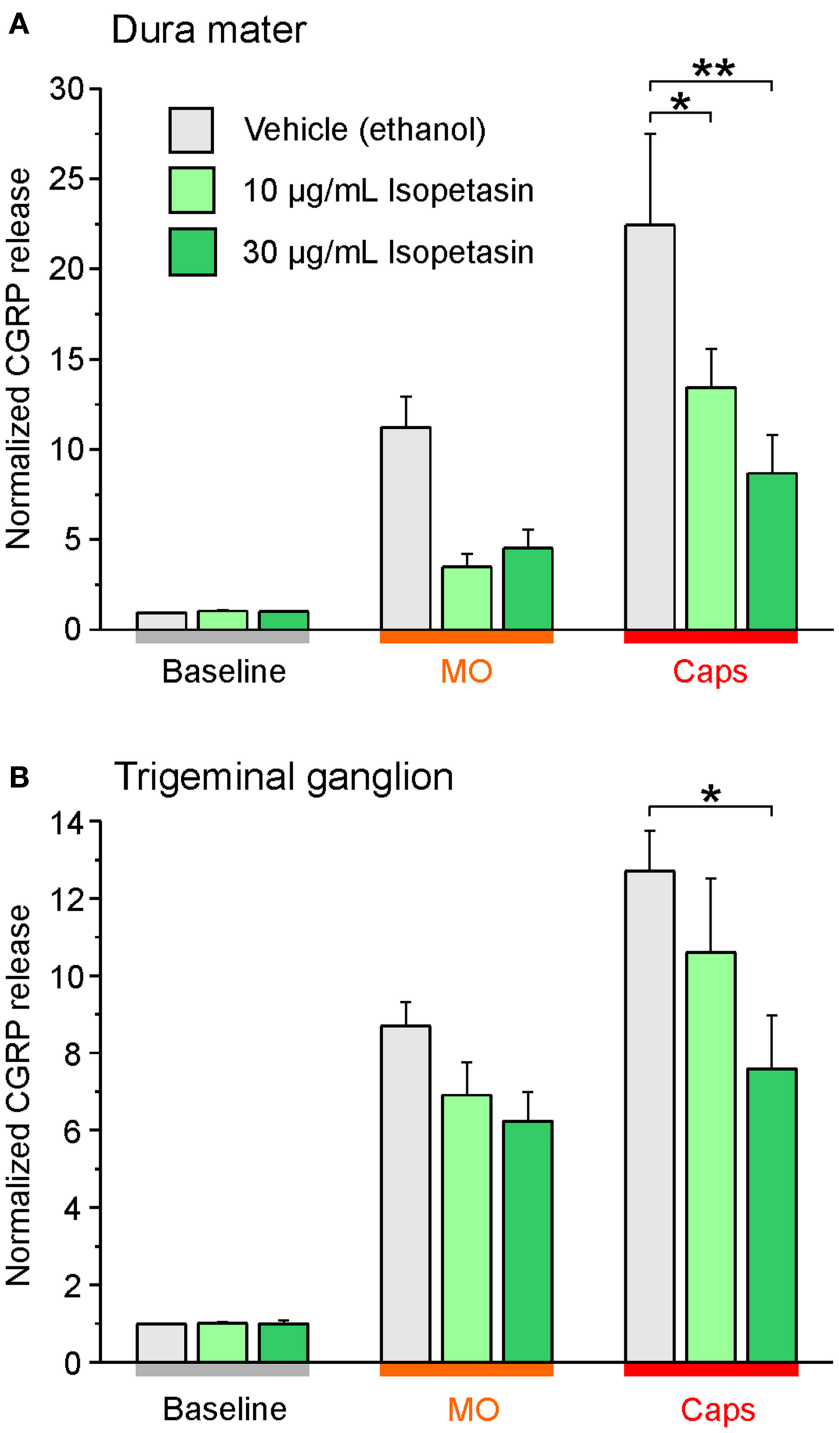
Isopetasin inhibits CGRP release from the dura mater **(A)** and trigeminal ganglia **(B)** of Wistar rats. Sagittally sectioned skulls freed of brain tissue but with adhering dura mater were incubated with isopetasin at concentrations of 10 and 30 μg/mL for 1 h (*N* = 6 independent experiments), and the data were compared to vehicle control treatments (ethanol, *N* = 12 independent experiments). CGRP release was stimulated with 5 x 10^−4^ M mustard oil (MO) and 5 x 10^−7^ M capsaicin (Caps); *p < 0.05, **p < 0.01, ANOVA and LSD *post-hoc* test. **(A)** dura mater; **(B)** trigeminal ganglion.

Next to studies with the butterbur root extract the inhibitory effect of isopetasin on CGRP release was investigated. Following stimulation with capsaicin the CGRP release from the dura mater was reduced at a concentration of 30 μg/mL isopetasin (Figure 5A). Alike, with trigeminal ganglion preparations, the differences in CGRP release between vehicle and the isopetasin treatments were significant after stimulation with the TRPV1 agonist capsaicin (Figure 5B). Collectively, the effects of butterbur extracts containing petasins and isopetasin on CGRP release are similar; however, the stronger effect seen with the butterbur extract at a concentration of 10 μg/mL is suggestive for other constituents in the root extract to be active as well.

**Figure 6:**
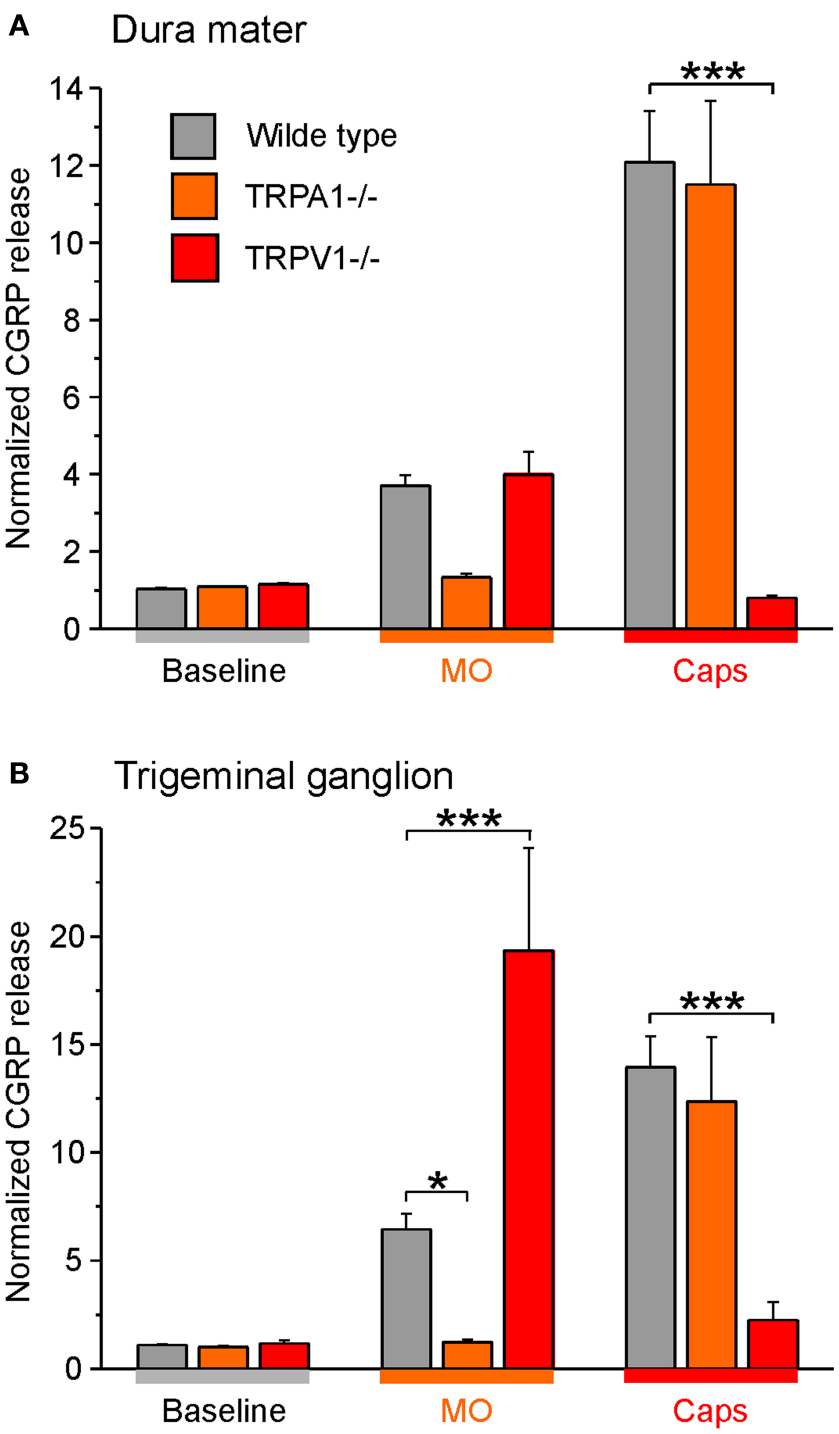
CGRP release from the dura mater **(A)** and trigeminal ganglia **(B)** of TRP -/- mice compared to C57 BL/6 wild type mice. Tissues were incubated with 30 μg/mL butterbur extract for 1 h. CGRP release was stimulated with 5 x 10^−4^ M mustard oil (MO) or 5 x 10^−7^ M capsaicin (Caps). *p < 0.05, ***p < 0.001, ANOVA and LSD *post-hoc* test.

Besides, studies with C57BL/6 wild-type mice and mice with functional knockout of TRP ion channels (-/-) were conducted. These experiments served as additional controls and demonstrated the role of the ion channels in the release of CGRP from dense core vesicles. As expected, TRPA1 receptor -/- mice did not respond to mustard oil stimulation with CGRP release from the cranial dura mater but stimulation with capsaicin increased the CGRP release significantly. Similar findings were obtained for wild-type mice (Figure 6A). Moreover, the dura mater of TRPV1 receptor -/- mice was stimulated with capsaicin which did not influence CGRP levels while mustard oil evoked increased CGRP release like in wild-type mice (Figure 6A).

Likewise, trigeminal ganglia of TRPA1 receptor -/- mice did not respond to CGRP release after stimulation with mustard oil whereas CGRP release was significantly increased after stimulation with the TRPV1 receptor agonist capsaicin (Figure 6B). Moreover, trigeminal ganglia of TRPV1 receptor -/- mice did not respond to stimulation with capsaicin but stimulation with mustard oil induced CGRP release drastically (Figure 6B). Notwithstanding, wild type mice responded to both treatments.

Together, the data suggest that the Petadolex butterbur root extract desensitizes nociception by acting on both TRPA1 and TRPPV1 ion channels.

### Clinical Efficacy of Petasites in Migraine

Petadolex® is the only butterbur extract that was clinically evaluated for its therapeutic benefit in the preventive treatment of migraine. The duration of treatment ranged from 3 to 4 months. The pivotal study by Lipton et al. (58) evaluated 4 months of treatment with Petadolex® in doses of 50 mg bid or 75 mg bid in a randomized, double-blind, placebo-controlled trial. The study included 233 patients with episodic migraine. Compared with placebo, the frequency of migraine attacks decreased in study participants receiving a daily dose of 150 mg Petadolex®. However, the difference from placebo was not statistically significant for the 100 mg dose (48% reduction for 150 mg, 36% reduction for 100 mg and 26% in the placebo group). The proportion of patients with a ≥50% reduction in attack frequency at 4 months was 68% for patients receiving Petadolex® at 150 mg, 56% in the 100 mg arm and 49% for the placebo arm (*p* < 0.05 for 150 mg Petadolex® vs. placebo).

A second study, designed as a randomized, group-parallel, placebo-controlled, double-blind trial, investigated the therapeutic efficacy of Petadolex® in 60 patients with episodic migraine. The patients received Petadolex® at a dosage of 2 capsules twice daily over 12 weeks (each capsule contains 25 mg Petadolex®). Inclusion criteria were a minimum of three attacks per month within the last 3 month prior to study enrolment in addition to a minimum of two attacks in the run-in phase, i.e., 4 weeks without trial medication. Subsequently, the patients were on butterbur (100 mg butterbur extract daily) for 12 weeks, and reductions in migraine attacks per 4 weeks defined the primary efficacy variable. Additionally, the patients reported the number of migraine days and the duration and intensity of migraine attacks. The clinical investigators reported high efficacy and excellent tolerance (61), and an independent reanalysis of the data by Diener et al. (62) revealed the 100 mg dose of Petadolex® to be effective. The ≥50% responder rate for migraine frequency was significantly higher in the Petadolex® study arm as compared to the placebo group (45 vs. 15%).

A third study, designed as prospective, randomized, partly double-blinded, placebo-controlled and parallel-group, investigated the efficacy of Petadolex® in 63 elementary school children with episodic migraine. The inclusion criteria were ages between 8 and 12 years, initial onset of migraine at least 1 year before study enrolment and an average of ≥2 attacks/month for the last 3 month. The study evaluated the efficacy of 50 to 150 mg daily doses (*N* = 19), music therapy (*N* = 20) or placebo (*N* = 19) for 12 weeks (63). Only music for therapy was superior to placebo (*p* = 0.005), while during the follow-up period both music therapy and butterbur extract were superior to placebo (*p* = 0.018 and *p* = 0.044, respectively). All groups showed a significant reduction in attack frequency relative to baseline.

### Clinical Trials With Butterbur Leaf Extracts in Other Indications

Given its anti-inflammatory properties butterbur extracts were also evaluated in conditions of seasonal allergic rhinitis (SAR). A clinical study investigated the effectiveness of butterbur extracts on nasal provocation testing in SAR. Twenty patients with grass-pollen-sensitized SAR were randomized in a double-blind, cross-over design to receive either 50 mg butterbur extract or placebo twice daily for 2 weeks during the grass pollen season. The study participants were challenged with a single dose of 400 mg/mL adenosine monophosphate (AMP), and the spontaneous recovery following AMP challenge defined the primary outcome. In this study butterbur significantly attenuated (*P* = 0.028) the peak nasal inspiratory flow from baseline following AMP challenge. Therefore, butterbur exhibited protection against AMP-induced nasal responsiveness during the grass pollen season in sensitized patients (64).

Additionally, sixteen patients with perennial allergic rhinitis and house dust mite sensitization were randomized in a double-blind, cross-over study. The study evaluated 50 mg butterbur bid, fexofenadine 180 mg once daily and placebo for 1 week (65). Both butterbur and fexofenadine were equally effective in attenuating the nasal response to AMP and improved nasal symptoms significantly.

Moreover, a prospective, randomized, double-blind, placebo-controlled, parallel-group compared the efficacy of a high (*N* = 60 patients) and low dose (*N* = 65 patients) butterbur leaf extract Ze339 to placebo (*N* = 61 patients). Established diagnostic criteria for intermittent allergic rhinitis were confirmed by skin allergy tests, and the extract was reported to be an effective treatment for intermittent allergic rhinitis (IAR) and was well tolerated (66). However, butterbur failed to demonstrate clinical efficacy in IAR in a smaller trial of 35 patients. Here, the patients received 50 mg butterbur, bid or placebo for 2 weeks, and the primary outcome variables were peak nasal inspiratory flow, nasal and eye symptoms and rhinoconjunctivitis-specific quality-of-life score (67). Notwithstanding, in a larger clinical trial of 330 patients designed as prospective, randomized, double-blind, parallel group comparison of butterbur leaf extract (Ze 339; 8 mg total petasin; one tablet thrice-daily) against fexofenadine (Telfast 180, one tablet once-daily) and placebo clearly established butterbur and fexofenadine as equally effective (68). Finally, butterbur conferred anti-inflammatory activity in atopic asthmatic patients maintained on inhaled corticosteroids (22).

## Pharmacokinetics

### Studies With the Caco-2 Cell Line and Rat Intestinal Segmental Uptake of Petasins

The absorption and metabolism of constituents of Petasites hybridus leaf extract Ze 339 (petasin content = 36.7%) were investigated in the human intestinal cell line Caco-2 and in anesthetized rats following intestinal instillations. The extract was dissolved in polyethylene glycol 400, and the apical to basolateral transport and reverse transport depended on the petasins examined and ranged between 9.4–17.5% and 3.3–6.7% in the Caco-2 cellular assay (69). In order to investigate the intestinal absorption in living animals the herbal extract solution was instilled into ligated intestinal segments (duodenum, jejunum, ileum), and blood samples were withdrawn from the portal vein at 0, 30, 60, 120 and 180 min. The uptake differed for the various petasins and petasols with highest absorption reported for the jejunum, followed by the ileum and duodenum nonetheless appeared to be dose proportional for the two doses evaluated.

### Human Pharmacokinetic Study

A study with human volunteers investigated plasma pharmacokinetics of petasins after oral administration of a single dose of 232 mg Petasites hybridus leave extract Ze 339, which corresponds to a dose of 32 mg petasin (70). The authors reported a Cmax for petasin of 58.1 +/- 26.7 ng/mL. Unfortunately, the study was only reported in a monograph proceeding, and validation of the bioanalytical assay (enzyme-linked immunosorbent assay, ELISA) was not shown.

### Modulation of CYP3A4 Monooxygenase Activity by Petasins

CYP3A4 monooxygenase is a major drug metabolizing enzyme, and its inhibition may influence drug clearance from the body. In order to identify concentrations that inhibit its activity studies with recombinant human CYP3A4 expressed in the V79MZh3A4hOR cell line were carried out. Here, an IC_50_ and IC_90_ of 0.019 and 2 mg/mL butterbur extract were determined. Given that the assays were performed with an extract containing 20.1% petasin, an IC_50_ for petasin of 3.8 μg/mL can be assumed which corresponds to >65-fold therapeutic Cmax petasins plasma concentration.

Conversely, a GLP compliant study with primary human hepatocyte (PHH) cultures evidenced significant increases in the 6β-hydroxylation of testosterone (71, 72). This metabolite signifies CYP3A4 catalytic activity and was induced after repeated treatments of PHH cultures at 93-fold therapeutic Cmax petasin plasma concentration. The results obtained with PHH cultures contrast the findings obtained with the V79MZh3A4hOR cell line described above.

Notwithstanding, the studies support the notion of petasins to modulate CYP3A4 activity; however, it was only observed at supra-therapeutic concentrations which is of no clinical relevance at the therapeutic dose of 3 mg/kg.

## *In vitro* Drug Interaction Studies With Primary Human Hepatocytes

To assess the potential of drug-herb interactions an *in vitro* study was performed, and liver function tests were evaluated in cultures of PHH treated with different butterbur extracts and medications commonly used for the treatment of migraine attacks (72). Additionally, CYP monooxygenase activities were evaluated using testosterone as a substrate. No hepatotoxicity was observed at therapeutic Petadolex® or Paracetamol, Ibuprofen and/or Naratriptan Cmax drug concentrations as shown by the MTT, LDH, ALT, γGT and urea assay. These assays monitor the reduction of the tetrazolium salt (3-(4,5-dimethylthiazol-2-yl)-2,5-diphenyltetrazolium bromide (MTT) by succinate dehydrogenase, the activity of lactate dehydrogenase (LDH), of alanine aminotransferase (ALT), gamma glutamyltransferase (γGT) and measure the production of urea.

However, at an excessive, i.e., >49-fold Cmax serum petasins and therapeutic Ibuprofen, Paracetamol and Naratriptan drug concentrations, slight to moderate changes in liver transaminase and CYP-monooxygenase activities were observed among individual hepatocyte donors as was reported by one of the authors of this review (72).

## Toxicology

Petadolex® is the only butterbur extract which has been extensively examined in preclinical safety studies. A total of 4 GLP compliant toxicity studies were carried out and include acute and repeated dose (28-days and 6-months) toxicity studies in rats (71). The extracts were administered parenterally and orally at dose levels up to 1200-fold of the maximal clinical dose (MCD = 3 mg/kg).

### Acute Toxicity

Acute toxicity of an excessive single i.p. dose of Petadolex® was evaluated in rats (71, 72). A median lethal dose of LD_50_ at 2,490 mg/kg was estimated (time frame of 14 days), and the doses examined ranged from 830 to 1245-fold of the MCD. No mortality and no signs for liver pathology were observed after a single i.p. injection of 490 mg/kg Petadolex® (=163-fold MCD).

### Repeated Dose Toxicity Study (28 Days)

In a 28-day dose range finding study clinical symptoms at high treatment doses, i.e., 0.90 and 1.80 g/kg, included salivation, reduced activity, squatting and stereotypic behavior (=intense, perseverative movements of the head, snout and orofacial area). Reduced food consumption and body weight gains were particularly obvious among male animals. Severe clinical symptoms and mortality in one female occurred in the highest dose group (corresponding to 600-fold MCD which is equivalent to 1,800 capsules swallowed once daily). Correspondingly, the highest treatment dose was reduced to 1.35 g/kg. Main findings in the 28-days dose range finding study were reduced organ weights of the kidneys and increased organ weights of the livers. The most significant histological findings were dose-related bile duct proliferations, which occurred in all treatment groups but were less severe among females (71, 72).

Blood smears revealed dose dependent increases in thrombocyte count as well as an reduction of hemoglobin and haematocrit levels in all treatment groups. In addition, liver function tests, i.e., glutamate dehydrogenase (GLDH), bilirubin and γGT, were significantly increased in the 0.90 and 1.35 g/kg-treatment groups, while aspartate aminotransferase (AST) and alkaline phosphatase (AP) were increased in some males at doses of 0.90 and 1.80 g/kg.

**Figure 7:**
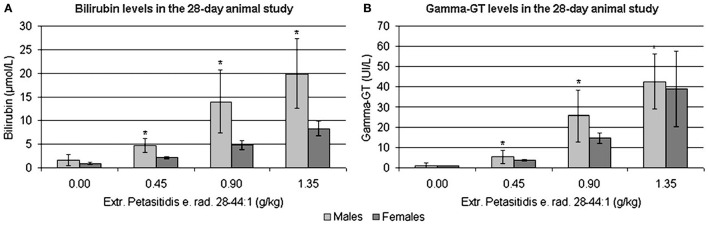
**(A)** Bilirubin (μmol/L) and **(B**) γGT levels (U/L) in rat serum treated with Extr. petasitidis e. rad. spiss. (28-44:1) containing <15% petasines, quantified as isopetasin for 28-days at various dose levels in a dose range finding study [taken from Anderson et al. (71)]. *p < 0.05.

Shown in Figure 7 are the dose-related changes in the serum biochemistries of bilirubin and γGT transaminase. Additionally, the laboratory measurements revealed mildly increased total plasma protein and albumin levels (≤ 5%) and reduced glucose and triglycerides by about 20 and 40%, respectively. The fact that Petadolex® lowered blood glucose and triglyceride levels is an important finding and points to an additional therapeutic use in the treatment of metabolic diseases. Indeed, an independent study reported petasins to lower blood glucose levels by stimulating the activity of adenosine monophosphate (AMP) -activated protein kinase which senses AMP/ATP intracellular levels and protects cells from adenosine triphosphate (ATP) deficiency (73).

### Repeated Dose Toxicity Study (6 Months)

As part of a GLP compliant toxicology study doses of up to 150-fold MCD were given by oral gavage in mygliol (an oily vehicle of medium chain triglycerides) for a period of 6 months (71, 72). In the course of the chronic toxicity study mortality was observed in the mid dose group (1 male, 2 females out of 40 animals) and the high dose group (3 males, 7 females out of 60 animals) but was also observed among control animals (3 males, 1 female out of 60 animals). Correspondingly, the high dose was reduced to 60-fold of the human MCD which is equal to 180 capsules swallowed once daily. Body weight changes occurred at mid and high doses and were decreased in males but increased in females and reversed to normal in a satellite recovery group subsequent to the 6-month treatment period. Absolute and relative organ weights of the liver and kidneys were dose-related increased in females at the mid and high doses. In males absolute and relative liver organ weights were increased at the high dose whereas relative kidney weights were significantly increased in all dose groups. The major histopathological findings for the liver were bile duct proliferations at 45-fold and bile duct dilatation at 90-fold of MCD with male rats being more frequently affected. However, histopathology did not reveal signs for necrosis, cholestasis or mixed type toxic liver injury. Furthermore, mild lipidosis was seen in vehicle treated control and high dose treated animals. The bile duct proliferation were complemented by mild increases in plasma bilirubin and γGT but there was no clear pattern in the temporary alterations of serum biochemistries during the 6-month chronic toxicity study with very slight but statistically insignificant changes of AST and ALT transaminases. In fact, these were reduced to approximately 90% of control values at the end of the study. While bilirubin was insignificantly increased by 4% in females and 85% in male rats there was considerable variability amongst individual animals. Importantly, the bile duct proliferations reversed, at least in part, during the recovery period as shown for a satellite group of animals subsequent to their 6 month treatment period (72).

Additional findings included a significant increase in thrombocyte count in males. Furthermore, prothrombin time was mildly but significantly increased at the low and mid dose in female animals after 6 months of treatment. The blood smears also indicated toxic granulation in some lymphocytes in male and female animals at the mid and high doses after 6 weeks and thereafter occurred in all treatment groups, including the controls.

Male rats presented basophilic and dilated protein-filled outer medullar tubules in kidneys. Immunostaining established the presence of alpha2microglobulin in the proximal convoluted tubules, and the morphological changes can be defined as alpha2microglobulin nephropathy. This lesion is found in male rats only in response to a wide range of drugs and chemicals; however, it has never been reported in patients and is of no clinical relevance (74). Histology also informed on regenerating renal tubular epithelial cells. The kidney findings were corroborated by increased urinary protein excretion and increased serum creatinine kinase activity; both returned to normal during continuous exposure.

Together, repeated dose toxicity studies with rats provide conclusive evidence for Petadolex® to be safe at therapeutic doses. Moreover, the chronic toxicity study provided evidence for adaptation during continuous exposure. Liver function tests and other serum biochemistry changes returned to normal during the course of the 6-month chronic toxicity study or in the subsequent recovery group, therefore demonstrating reversibility of the findings. A no effect level (NOAEL) for Petadolex® was established at 45 mg/kg that is equivalent to 15-fold MCD or 300 capsules given once daily.

**Figure 8:**
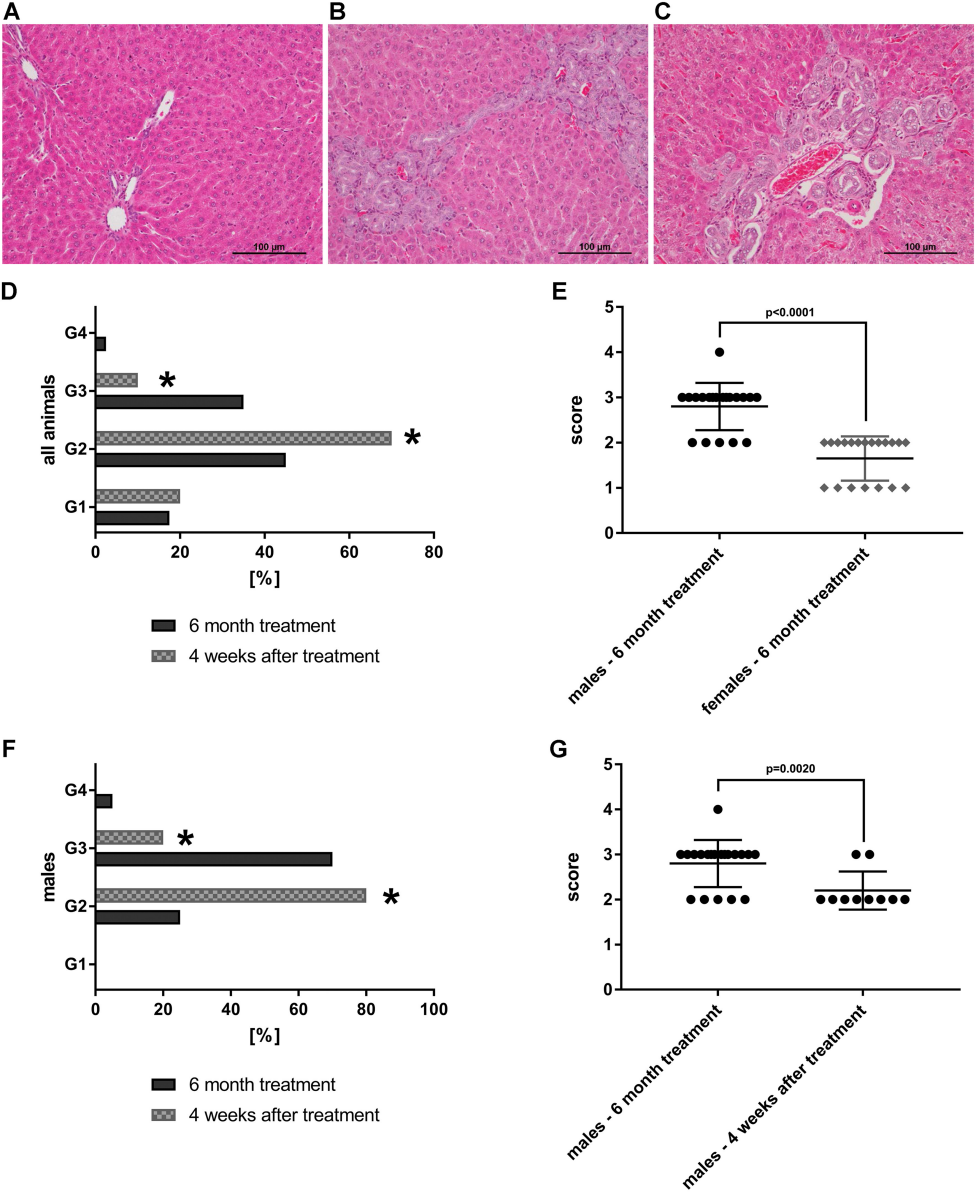
HE-stained histopathological slices of livers from SD-rats treated with petasites hybridus extracts (with at least 15 % petasin quantified as isopetasin). *Biliary dilatations and biliary duct hyperplasia in periportal fields were observed after 6 months of treatment*. **(A)** Control recovery group; **(B,C)** high dose group (0.270 g/kg). The liver sections were examined under a light microscope (Nikon Ni-E microscope, Japan) images were captured and documented with the Nikon NIS basic research microscopic imaging software version 4.3. The bar represents 100 μm. *Bile duct proliferations after repeated oral dosing of rats with a Petasites hybridus extract (Petadolex®) for 6 months and a recovery period of 4 weeks*. Bile duct proliferations were dosage related increased. The grading of lesions ranges from minimal (G1), mild (G2), moderate (G3) to marked (G4). **(D)** Grading of bile duct proliferations of 40 high dose animals (dark column) and 20 recovery animals (gray patterned) treated for 6 month. Using a Fisher exact test, a statistically significant reduction from moderate (G3) to mild (G2) was calculated for animals which were allowed to recover from the treatment for 4 weeks (*p* = 0.0001). **(E)** Sex related differences in bile duct proliferations after repeated treatment of rats for 6 months. Males are more sensitive to the treatment effects as determined by the Wilcoxon-Mann-Whitney-Test. **(F)** Grading of bile duct proliferations of 20 high dose males (dark column) and 10 male recovery animals (gray patterned) treated for six months. Using the Fisher exact test, a statistically significant reduction from moderate (G3) to mild (G2) was calculated for animals that were allowed to recover from the treatment for 4 weeks (*p* = 0.0161). **(G)** The scores for male animals after repeated treatment for 6 months and male recovery animals are compared. The grades of lesion differed significantly as determined by the Wilcoxon-Mann-Whitney-Test. The data shown in panel d-g were taken from Anderson and Borlak (72). *Statistical significant with a *p*-value of < 0.05.

Shown in Figure 8 are liver sections from control (panel A) and petasites hybridus extract treated animals at the high dose, i.e. 90-fold MCD, after daily dosing for 6 months (panel B&C). Additionally, we compared the histological scores in animals that recovered from the treatment for a period of 4 weeks (“recovery animals”, panel D, F, G). The grading of lesions ranged from minimal (G1), mild (G2), moderate (G3) to marked (G4), and we observed a significant difference between male and female animals (panel E, *p* < 0.0001). Male animals are more sensitive to the petasites hybridus extract and the bile duct proliferations were dosage related. Nonetheless, the bile duct proliferations tended to be reversible, and a statistically significant reduction from a mean moderate (G3) to mild (G2) was determined in male animals at the high dose using the Fisher exact test (*p* = 0.0020, panel F&G).

## Genotoxicity

Petasites hybridus plants vary in their PA content. Experimental and clinical evidence established PAs to be harmful and potentially carcinogenic for their metabolic activation to reactive metabolites by CYP450 monooxygenases (75–77). Currently >600 PAs have been identified among thousands of plants, and because of their potency and genotoxicity the 1, 2-unsaturated PAs are of particular concern. To this effect the German Federal Institute for Risk Assessment (BfR) prepared a white paper on the health risks associated with PA contaminated food and herbs (78). Specifically, metabolic activation converts PAs into pyrrolic electrophiles which bind to DNA, damage nucleic acids and cause mutations.

The genotoxic potential of PAs is similar among those with an unsaturated necine ring and one or two ester groups in the molecule. Senecionine and its N-oxide are the main PAs in root extracts of Petasites hybridus, followed by integerrimin and senkirkin (15). The genotoxic potential of Petadolex® was investigated in a GLP compliant Ames test in the presence and absence of a liver homogenate for their metabolic activation (S9 mix). Alike, its genotoxic potential was evaluated in a GLP compliant chromosomal aberration assay with human lymphocytes. In all of these studies no mutagenic effect was observed (79, 80).

Since PAs are constituents of Petasites hybridus, the manufacturer of Petadolex® developed a supercritical CO_2_ extraction method to remove PAs to levels close to the limit of detection. Although guided by precaution, the regulatory authorities argued that this change in the extraction method would alter the composition of the extract which therefore is not the same as the original one that received market authorization [the regulatory issues are addressed below, see also Diener et al. (81)].

Based on the toxicological threshold concept (TTC) for potentially human carcinogens a maximum daily dose of 1.5 μg/day PA containing medications may be considered as acceptable. The European guideline on genotoxic impurities (EMEA/CHMP/QWP/251344/2006) requires a ten-fold lower limit, i.e., 0.15 μg/day for high potency carcinogens. The manufacturer of Petadolex® specifies the total PA content to ≤ 0.035 ppm for a daily therapeutic dose of 150 mg Petadolex®. Therefore, a worst case scenario would result in an exposure of 0.005 μg total PAs which is 300-fold less than the limit set by the regulatory authorities. Collectively, the risk from genotoxic impurities is negligible based on the limit set for high potency carcinogens.

## Butterbur Regulates Bile Salt Transporters

The genomic study with PHH cultures provided a molecular rationale for bile duct proliferation seen in the rat chronic toxicity study (71). Excessive butterbur concentrations (49-fold Cmax therapeutic petasin plasma concentrations) caused induction but also repression of diverse bile salt transporters and other solute carriers in cultures of human hepatocytes. Specifically, the ATP binding cassette subfamily B member 1 (ABCB1) transporter was significantly upregulated in PHH, and this is also observed in rats after bile duct ligation due to bile flow obstruction (82). ABCB1 has broad substrate specificity, and its expression is largely dependent on the activity of the Farnesoid X (FXR) bile acid receptor (83). Thus, butterbur influences the FXR/retinoid X receptor signaling pathway in human hepatocytes. The genomic study also revealed ABCB4 (Mdr2/MDR3) to be repressed. This canalicular membrane transporter plays an essential role in the biliary excretion of phospholipids. The sodium/taurocholate co-transporting polypeptide (SLC10A1, repressed at >49-fold therapeutic Cmax petasin plasma concentrations) functions in bile acid uptake, and its regulation is an adaptive response to protect hepatocytes against toxic intracellular bile acid accumulation. Together, 18 solute carriers were regulated in human hepatocyte cultures and provide a rationale for adaptive responses to impaired bile acid transport and associated bile duct proliferations in rats at 45 and 90-fold MCD.

Additionally, the genomic study revealed the solute carriers Slc17a1and Slc17a3 to be induced in rat hepatocyte cultures. Both are type I Na+-dependent phosphate transporter and are localized at the sinusoidal membrane and function as bile acid co-transporters. Furthermore, the sodium/bile acid cotransporter Slc10a2 was significantly repressed; this sodium taurocholate co-transporting polypeptide is a major transporter of conjugated bile acids (84). Collectively, at excessive and well above therapeutic doses butterbur caused altered bile acid homeostasis with the regulation of diverse bile salt transporter.

## Regulatory Issues

The following regulatory issues require consideration and were described in more detail for the product Petadolex® in the review of Diener et al. (81). Specifically, in 1988 the manufacturer of Petadolex® changed the extraction procedure from the solvent methylene chloride to CO_2_ to improve the removal of PAs. Note, all published studies establishing clinical efficacy of Petadolex® were performed with the CO_2_ extract as this is the sole extract marketed in the USA and Canada. In 2002, the German regulatory authority BfArM assessed this modification in the extraction protocol and concluded that this change had altered the composition of active ingredients. BfArM considered Petadolex® not to be the original one for which market authorization was granted, and Petadolex® was removed from the market. However, PAs do not contribute to the pharmacological activity of the product but are a safety concern. Given that the specification of the active ingredient remained the same the decision to withdraw market authorization is perplexing. In addition, the manufacturer of Petadolex® applied for a new traditional license of the UK Medicines and Healthcare products Regulatory Agency (MHRA) which was declined because of safety concerns related to the distribution by general sale outlets. Notwithstanding, a product license for Petadolex® was issued by Health Canada in 2021.

A further point of considerable concern is the lack of product label specifications among the different butterbur brands. Specifically, provisions of federal law in the US require nutrition labeling only but information on the amount of active ingredient is not a legal requirement nor are manufacturers required to supply information on potential hazardous impurities and their quantities. Importantly, an analysis of 21 commercial butterbur products by the University of Mississippi, National Center for Natural Products Research, revealed that the majority of these products do not specify the quantity of their ingredients but contain PAs which is an important safety concern (85). Therefore, regulators should request manufacturers to provide the specifications of toxic ingredients in butterbur extracts.

## Conclusion

Butterbur is effective in the prevention of migraine attacks, and recent research is highly suggestive for a mechanism whereby petasins block CGRP nociceptive pathways. However, only one product (Petadolex®) was extensively evaluated in preclinical and clinical studies in migraine to demonstrate therapeutic efficacy and safety. Given the many butterbur brands and uncertainties regarding their petasin content, the need for labeling of products remain an important topic. Furthermore, manufacturers should be requested to inform patients on potential harmful ingredients such as pyrrolizidine alkaloids. Based on information provided by the manufacturer as well as independent investigations Petadolex® is essentially free of PA contaminants. In regards to butterbur safety, the spontaneous reports on hepatobiliary events in relation to Petadolex® use have been evaluated (*N* = 48 cases over a period of > 30 years and an estimated 2.6 million patient month exposure) (72). The causality assessment showed most cases to be confounded by hepatotoxic co-medications especially during periods of pain. Notwithstanding, in clinical trials with migraine patients there was not a single case with clinically relevant liver function abnormalities.

Collectively, butterbur offers an alternative treatment for migraine therapy, and future research will identify which of the petasins are best in blocking CGRP dependent transmission of nociceptive information. Butterbur extracts reduce CGRP release from trigeminal afferents by desensitizing TRPA1 and TRPV1 receptor channels.

Finally, whether patients failing on antibody based or gepants treatments would benefit from butterbur based therapies in reducing migraine attacks is an open question that needs to be addressed.

## Author Contributions

JB wrote the final manuscript. All authors contributed to writing—review and editing this manuscript.

## Conflict of Interest

JB: The manufacturer of the butterbur extract Petadolex®, Weber & Weber GmbH & Co. KG, Germany funded a genomic and mechanistic safety study during the period 2008–2010. Currently, Weber & Weber GmbH & Co. KG sponsors an investigator initiated study on CGRP receptor signaling pathways and JB serves as a scientific advisor and receives a consultancy fee. He also receives funding from the German Ministry of Education and Research (BMBF) and the European Union, the Food and Drug Administration of the US and is a member of the editorial board of the BMC Journal Genome Medicine and the Journal of Clinical and Translational Hepatology. H-CD received honoraria for participation in clinical trials, contribution to advisory boards or oral presentations from: Lilly, Novartis, Pfizer, Teva and Weber & Weber. The German Research Council DFG, the German Ministry of Education and Research BMBF and the European Union support his headache research. H-CD serves on the editorial boards of Cephalalgia and Lancet Neurology and member of the Clinical Trials Committee of the IHS. KM received honoraria and/or financial support for traveling and housing from the Lilly GmbH, Novartis Pharma GmbH, Pharm-Allergan GmbH and Teva GmbH/AG and consulting honoraria from Amgen Inc. and Teva Pharmaceuticals. The research projects of KM were supported by Allergan Holdings Ltd., the Alexander von Humboldt Foundation, the European Union, Merck & Co., Inc, Teva Pharmaceuticals and the Weber & Weber GmbH & Co. KM is a member of the editorial board of Cephalalgia. The remaining authors declare that the research was conducted in the absence of any commercial or financial relationships that could be construed as a potential conflict of interest.

## Publisher's Note

All claims expressed in this article are solely those of the authors and do not necessarily represent those of their affiliated organizations, or those of the publisher, the editors and the reviewers. Any product that may be evaluated in this article, or claim that may be made by its manufacturer, is not guaranteed or endorsed by the publisher.

## Abbreviations

ABCB1: ATP binding cassette subfamily B member 1
AITC: allyl isothiocyanate
ALT: alanine aminotransferase
AMP: adenosine monophosphate
AP: alkaline phosphatase
AST: aspartate aminotransferase
ATP: adenosine triphosphate
Ca^2+^: calcium ions
CGRP: calcitonin gene related peptide
COX-2: cyclooxygenase-2
CYP: cytochrome
BfArM: Bundesinstitut für Arzneimittel und Medizinprodukte
FXR: farnesoid X receptor
γGT: gamma glutamyltransferase
GLDH: glutamate dehydrogenase
GLP: Good Laboratory Practice
HE stain: hematoxylin and eosin stain
HET-CAM test: hen's egg-chorioallantoic membrane test
IC_50_/IC_90_: inhibitory concentration values of drugs
LD_50_: Lethal Dose 50
LDH: Lactate dehydrogenase
LPS: lipopolysaccharide
LSD: least significant difference
LTB4: leukotriene B4
LTE4: leukotriene E4
LTs: cysteinyl-leukotrienes
MAPK: mitogen-activated protein kinases
MCD: maximal clinical dose
MDR2/MDR3: multidrug resistance-associated protein
MHRA: Medicines and Healthcare products Regulatory Agency
MRP2: multidrug resistance-associated protein 2
MTT: tetrazolium salt (3-(4, 5-dimethylthiazol-2-yl)-2,5-diphenyltetrazolium bromide
NMRI mice: Naval Medical Research Institute inbred mice
NO: nitric oxide
NOAEL: no-observed-adverse-effect level
Pas: pyrrolizidine alkaloids
PGD2: prostaglandin D2
PGE2: prostaglandin E2
PHH: primary human hepatocytes
SAR: seasonal allergic rhinitis
SLC10A1: solute carrier family 10 member 1
TRP (TRPA, TRPV): family of transient receptor potential cation channels transient receptor potential
TTC: toxicological threshold concept
VDCC: voltage-gated calcium channels.
